# Synthesis and pharmacological evaluation of guanidinederivatives with potential hypoglycemic activity

**DOI:** 10.1186/1753-6561-8-S4-P10

**Published:** 2014-10-01

**Authors:** Patrícia de Albuquerque Sarmento, Polliane Maria Cavalcante-Araújo, Igor Santana de Melo, Paulo Henrique Barcellos França, Návylla Candeia de Medeiros, Tales Lyra de Oliveira, Thaís Honório Lins, Regina Célia Sales Santos Veríssimo, Maria Lysete de Assis Bastos, Êurica Adélia Nogueira Ribeiro, Robinson Sabino-Silva, João Xavier de Araújo-Júnior

**Affiliations:** 1 Laboratório de Pesquisa em Recursos Naturais, Universidade Federal de Alagoas, Maceió, Brazil; 2 Laboratório de Neurociências e Fisiologia Integrativa, Universidade Federal de Alagoas, Maceió, Brazil; 3 Laboratório de Pesquisa e Tratamento de Feridas, Universidade Federal de Alagoas, Maceió, Brazil

## Background

The hyperglycemia characteristic of diabetes can cause cellular and tissue damage due to the biochemical alterations that lead to the formation and accumulation of advanced glycation end products (AGEs) [1]. Aminoguanidine (AG) prevents the formation of AGEs by reacting with the initial glycation products, proving to be effective in improving proteinuria and vessel elasticity, in the prevention of diabetic retinopathy, and in the treatment of patients with diabetic nephropathy [2]. Guanidine derivatives have demonstrated various biological activities, such as antihypertensive and antidiabetic effects. It has been proposed that aminoguanidine and some of its derivatives (DAGs) may increase sensitivity to insulin [3]. The aims of this study were to investigate the effect of AG and two of its derivatives (DAG11 and DAG15) on the regulation of blood glucose and on the insulin tolerance test (ITT) in normoglycemic and diabetic rats, and the possible toxic effects of these derivatives.

## Method

Wistar rats, 2 months of age, which had diabetes induced by Alloxan (40 mg/kg, i.v.), and their controls were administered the vehicle. At 21 days after the induction of diabetes, the animals were intraperitoneally treated for 7 consecutive days with saline (SAL), AG (10 mg/kg), DAG11 or DAG15 (10 mg/kg). On the 28th day the animals were anesthetized (ketamine, 80 mg/kg and xylazine, 12 mg/kg) and the ITT was performed with the administration of insulin (0.75 UI/kg, i.v.) for the analysis of insulin sensitivity through the glucose decay constant (klTT). The results were expressed as mean ± SEM and were compared using ANOVA, with Student-Newman-Keuls post hoc test (p ≤ 0.05). The toxicological evaluation was performed using human lymphocytes[4]. The study was approved by the Ethics Committee for the use of animals of the Federal University of Alagoas UFAL: 01/2012.

## Results and conclusions

The basal blood glucose of the animals treated with SAL, AG, DAG11 and DAG15 presented no significant differences. In the normoglycemic animals, although no significant difference occurred between the experimental groups, there was an increase of 55% and 67% in the kITT of the AG and DAG15 rats, respectively, compared to the SAL animals. The diabetic animals treated with AG and DAG15 presented increased (p ≤ 0.05) kITT (150% and 81%) compared to the SAL animals. No toxic effects were observed for any of the substances tested, at least not in the model used. Despite having no action on basal glucose, AG and DAG15 may be promising prototypes for diabetes treatment drugs, in view of their increased insulin sensitivity action in diabetic animals.
